# The effect of regularly dosed paracetamol versus no paracetamol on renal function in *Plasmodium knowlesi* malaria (PACKNOW): study protocol for a randomised controlled trial

**DOI:** 10.1186/s13063-018-2600-0

**Published:** 2018-04-24

**Authors:** Daniel J. Cooper, Katherine Plewes, Matthew J. Grigg, Giri S. Rajahram, Kim A. Piera, Timothy William, Mark D. Chatfield, Tsin Wen Yeo, Arjen M. Dondorp, Nicholas M. Anstey, Bridget E. Barber

**Affiliations:** 1 Global and Tropical Health Division, Menzies School of Health Research and Charles Darwin University, Darwin, NT Australia; 2 Infectious Diseases Society Sabah-Menzies School of Health Research Clinical Research Unit, Kota Kinabalu, Sabah Malaysia; 3 Mahidol Oxford Tropical Medicine Research Unit, Faculty of Tropical Medicine, Mahidol University, Bangkok, Thailand; 4 Centre for Tropical Medicine and Global Health, Nuffield Department of Medicine, University of Oxford, Oxford, UK; 5 Division of Infectious Diseases, Faculty of Medicine, University of British Columbia, Vancouver, Canada; 6 Infectious Diseases Unit, Clinical Research Centre, Queen Elizabeth Hospital, Kota Kinabalu, Sabah Malaysia; 7 Sabah Department of Health, Kota Kinabalu, Sabah Malaysia; 8 Jesselton Medical Centre, Kota Kinabalu, Sabah Malaysia; 9 Queensland Institute of Medical Research, Brisbane, Australia; 10 Lee Kong Chian School of Medicine, Nanyang Technological University, Singapore, Singapore

**Keywords:** Malaria, Plasmodium knowlesi, Acute kidney injury, Paracetamol

## Abstract

**Background:**

*Plasmodium knowlesi* is the most common cause of human malaria in Malaysia. Acute kidney injury (AKI) is a frequent complication. AKI of any cause can have long-term consequences, including increased risk of chronic kidney disease, adverse cardiovascular events and increased mortality. Additional management strategies are therefore needed to reduce the frequency and severity of AKI in malaria. In falciparum malaria, cell-free haemoglobin (CFHb)-mediated oxidative damage contributes to AKI. The inexpensive and widely available drug paracetamol inhibits CFHb-induced lipid peroxidation via reduction of ferryl haem to the less toxic Fe^3+^ state, and has been shown to reduce oxidative damage and improve renal function in patients with sepsis complicated by haemolysis as well as in falciparum malaria. This study aims to assess the ability of regularly dosed paracetamol to reduce the incidence and severity of AKI in knowlesi malaria by attenuating haemolysis-induced oxidative damage.

**Methods:**

PACKNOW is a two-arm, open-label randomised controlled trial of adjunctive paracetamol versus no paracetamol in patients aged ≥ 5 years with knowlesi malaria, conducted over a 2-year period at four hospital sites in Sabah, Malaysia. The primary endpoint of change in creatinine from enrolment to 72 h will be evaluated by analysis of covariance (ANCOVA) using enrolment creatinine as a covariate. Secondary endpoints include longitudinal changes in markers of oxidative stress (plasma F_2_-isoprostanes and isofurans) and markers of endothelial activation/Weibel–Palade body release (angiopoietin-2, von Willebrand Factor, P-selectin, osteoprotegerin) over 72 h, as well as blood and urine biomarkers of AKI. This study will be powered to detect a difference between the two treatment arms in a clinically relevant population including adults and children with knowlesi malaria of any severity.

**Discussion:**

Paracetamol is widely available and has an excellent safety profile; if a renoprotective effect is demonstrated, this trial will support the administration of regularly dosed paracetamol to all patients with knowlesi malaria. The secondary outcomes in this study will provide further insights into the pathophysiology of haemolysis-induced oxidative damage and acute kidney injury in knowlesi malaria and other haemolytic diseases.

**Trial registration:**

Clinicaltrials.gov, NCT03056391. Registered on 12 October 2016.

**Electronic supplementary material:**

The online version of this article (10.1186/s13063-018-2600-0) contains supplementary material, which is available to authorized users.

## Background

The simian parasite *Plasmodium knowlesi*, first reported as a major cause of human malaria in Malaysia in 2004 [1], is now known to cause human infections throughout Southeast Asia [2]. In Malaysia, *P. knowlesi* is the most common cause of human malaria, and incidence is increasing [3–5]. In Sabah, 1325 cases of knowlesi malaria were notified in 2014, representing a 33% increase on 2013 notifications [6]. Recent studies using more specific molecular diagnostic methods indicate that *P. knowlesi* is also the most common cause of human malaria in areas of western Indonesia [7, 8].

As with falciparum malaria, acute kidney injury (AKI) is a common complication of knowlesi malaria. Severe AKI (serum creatinine > 265 μmol/L) occurred in 24% of patients in a prospective tertiary-hospital study of severe *P. knowlesi* malaria [9] and in 54% of fatal knowlesi malaria cases in Sabah during 2010–2014 [6, 10]. Even in patients with non-severe knowlesi malaria, mild-moderate AKI is common. In a recent district hospital-based study involving 481 patients with knowlesi malaria of any severity, AKI by Kidney Disease: Improving Global Outcomes (KDIGO) criteria [11] occurred in 26% of children ≤ 12 years and in 19% of adults [12]. Importantly, AKI, even if not severe, can have significant long-term consequences. Regardless of cause, AKI is associated with increased risk of chronic kidney disease (CKD) [13], progression of existing CKD [14], long-term risk of end-stage renal disease [15], major adverse cardiovascular events [16] and mortality [13, 17]. In addition, AKI is associated with an increased duration of hospitalisation and need for specialised long-term care, thus contributing to substantial health economic costs [18]. Interventions aimed at reducing the incidence and severity of AKI are therefore needed, including appropriate adjunctive treatments targeting the pathogenic mechanisms underlying AKI.

Mechanisms of renal disease in knowlesi malaria are poorly defined. In falciparum malaria, AKI has been attributed in part to haemolysis-mediated oxidative damage [19]. Haemolysis, and subsequent release of cell-free haemoglobin (CFHb), leads to oxidative stress and lipid peroxidation in the renal tubules via oxidation of ferric (Fe^3+^) to ferryl (Fe^4+^) haemoglobin [20]. Free-radical-induced lipid peroxidation generates F_2_-isoprostanes (F_2_-IsoPs) and isofurans (IsoFs), which appear in free form in plasma and subsequently, after glomerular filtration, in urine. F_2_-IsoPs and IsoFs are considered robust measures of oxidative stress, and are associated with AKI in haemolytic conditions such as rhabdomyolysis, sepsis and post-cardiopulmonary bypass [20–24]. Similarly, in a prospective observational study of Bangladeshi adults with severe falciparum malaria, CFHb and F_2_-IsoPs/IsoFs were associated with AKI and disease severity, suggesting that haemolysis-induced oxidative damage contributes to the pathogenesis of malaria-associated AKI [19].

Paracetamol has been shown to inhibit CFHb-induced lipid peroxidation via reduction of ferryl haem to the less toxic Fe^3+^ state and quenching of globin radicals [20, 25, 26]. In a proof-of-concept study in rats, paracetamol significantly reduced rhabdomyolysis-induced kidney injury by inhibiting haemoprotein-catalysed lipid peroxidation [20]. In a retrospective study of patients with sepsis and raised CFHb, receiving paracetamol was associated with reduced lipid peroxidation and reduced risk of death [27]. In a randomised, placebo-controlled trial, paracetamol was associated with reduced plasma F_2_-IsoPs and improved renal function in adults with sepsis and detectable CFHb [28]. Paracetamol was also associated with decreased lipid peroxidation (plasma IsoFs) in adults [21] and children [23] undergoing cardiopulmonary bypass. A recent randomised controlled trial in Bangladeshi adults with severe and moderately severe falciparum malaria showed that paracetamol improved kidney function and reduced odds of developing AKI, particularly in those with high CFHb at enrolment [29].

Paracetamol may also have a potential role in reducing endothelial activation and microvascular dysfunction in malaria. In previous in vitro studies, CFHb has been shown to stimulate degranulation of endothelial cell Weibel–Palade bodies (WPBs), leading to release of major WPB constituents von-Willebrand factor (vWF) and P-selectin [30]. In addition, WPBs also store and release angiopoietin-2 (Ang-2), an autocrine mediator of endothelial activation that is associated with mortality and endothelial dysfunction in severe falciparum malaria [31], and with AKI in knowlesi malaria [32]. In both species, CFHb is independently associated with Ang-2 [32–34], suggesting that CFHb-induced degranulation of WPBs may also lead to release of Ang-2. The haem-mediated release of WPB constituents has been shown to be mediated through TLR4 signalling, and is dependent on NADPH oxidase (NOX) [30]. Paracetamol has been shown to reduce NADPH isoforms NOX2 and NOX4, the major NOX isoforms expressed in the kidney and linked to the pathogenesis of AKI [35]. Thus, prevention of CFHb-induced WPB release may represent an additional renoprotective mechanism of paracetamol.

### Rationale

In adults with severe knowlesi malaria, CFHb is higher than in severe falciparum malaria, and AKI at least as common [32]. As paracetamol has been shown to reduce haemolysis-induced lipid peroxidation, we hypothesise that paracetamol may play a similar role in patients with knowlesi malaria [19, 27, 33, 36]. Paracetamol is inexpensive, safe and widely available. If a renoprotective role is demonstrated, this would provide evidence for the administration of regular paracetamol for all patients with knowlesi malaria.

## Methods

We aim to test whether regularly dosed paracetamol compared with no paracetamol will reduce kidney dysfunction in patients with *P. knowlesi* malaria.

### Trial design and study sites

**Fig. 1:**
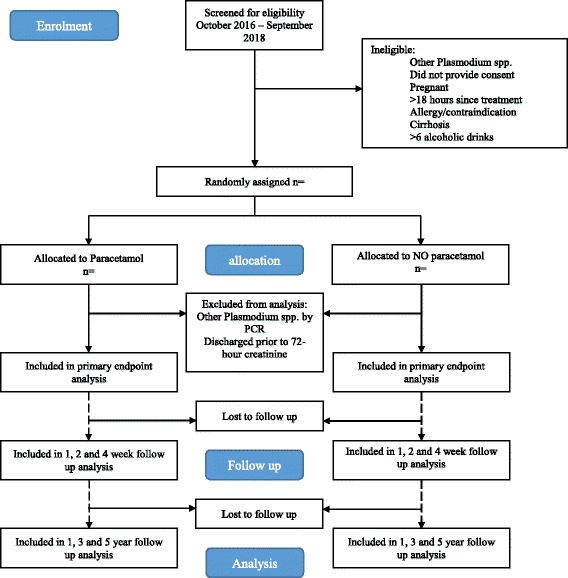
PACKNOW trial design

This is a two-arm, open-label, randomised controlled trial conducted at four centres in Sabah, Malaysia, namely Queen Elizabeth Hospital (Kota Kinabalu) and Keningau, Ranau, and Kota Marudu District Hospitals. Queen Elizabeth Hospital and Keningau both have well-equipped intensive care units with facilities for invasive ventilation, haemodynamic support and renal replacement therapy. Patients will be followed up at 1, 3 and 5 years post-enrolment. A summary of the trial design is shown in Fig. 1.

### Recruitment

Patients are being identified via referral from hospital physicians and/or notification from hospital microscopists. Patients are being recruited in Emergency Departments at time of presentation, or in medical wards or outpatient departments. Patient recruitment started in October 2016 and is expected to be complete by September 2018.

### Participants

Inclusion criteria:

1. Age ≥ 5 years
2. Admitted to hospital with microscopy-diagnosed *P. knowlesi* infection^1^
3. Temperature > 38 ºC on admission or fever during the preceding 48 h
4. Within 18 h of commencement of antimalarial treatment
5. Written informed consent from patient or attending relative/guardian

Exclusion criteria:

1. Contraindication or allergy to paracetamol or artesunate therapy
2. Known cirrhosis, or more than six standard alcoholic drinks per day
3. Pregnancy^2^

### Randomisation and blinding

Participants are randomised to either the paracetamol or the control arm using computer-generated site-specific block randomisation by an independent statistician in a 1:1 ratio. Allocation tables were uploaded to the Research Electronic Data Capture (REDCap) web application hosted at Menzies School of Health Research. REDCap is a secure, web-based application designed to support data capture for research studies [40]. Standardised clinical data is entered directly into the REDCap electronic case report form (CRF). Enrolment procedures do not delay administration of standard artemisinin-based treatment. As placebo tablets are not used in the no paracetamol arm, it is not possible to blind clinicians, investigators or patients to the treatment allocation. However, treatment allocation is masked from staff performing all laboratory investigations.

### Treatment arms

1. Paracetamol arm: Paracetamol is administered orally 6-hourly for 72 h by research nurses. Patients unable to swallow receive paracetamol by nasogastric tube. Dosing of paracetamol is weight-based, with patients ≥ 50 kg receiving 1 g and patients < 50 kg receiving 12.5–15 mg/kg. If the patient vomits within 30 min of paracetamol administration, a further dose is given.
2. No paracetamol arm (control): Patients in the control arm do not receive paracetamol; however, paracetamol may be given if temperature remains > 39.5 °C for > 30 min despite tepid sponging, or if deemed necessary by the treating clinician. There is no specific rescue medication for this trial.

The date, time and dose of paracetamol administered to patients is recorded in the CRF. All patients receive standard artemisinin-based treatment for malaria. The use of intravenous artesunate and oral artemisinin-combination therapy is at the discretion of the treating clinician according to Malaysian National guidelines [41].

Day 0, Hour 0 (enrolment) is the time of administration of the first dose of paracetamol administered by the research team. Any concomitant medications, or paracetamol doses prior to enrolment, are recorded on the CRF. There are no medications that are not permitted during the study; however, it is recommended to avoid non-steroidal anti-inflammatory drugs to avoid exacerbating any renal dysfunction.

Paracetamol 500 mg tablets administered in this study are produced locally in a Good Manufacturing Practice-qualified facility (*Paracil*™, SM Pharmaceuticals, Malaysia).

### Sample size

Our sample size for ANCOVA was calculated using the Stata command sampsi, with *α* = 0.05; 1 − *β* (power) = 0.9; log-transformed estimated mean 72 h creatinine in control group = 4.38 μmol/L (estimated SD 0.36); log-transformed estimated mean 72 h creatinine in treatment group = 4.275 μmol/L (estimated SD 0.36); correlation between baseline and 72 h creatinine (data log-transformed) = 0.59. The mean 72 h creatinine in the control group, the correlation between baseline and 72 h creatinine, and the standard deviation of the creatinine was estimated using existing data from prospectively enrolled patients with non-severe knowlesi malaria at a tertiary hospital in Sabah [9], with non-severe malaria patients used to more accurately reflect the patients at the district hospital sites. The mean 72 h creatinine in the treatment group was estimated using an estimated effect size of 10%, consistent with data from a pilot randomised trial of regularly dosed paracetamol, versus no paracetamol, in patients with severe and moderately severe falciparum malaria in Bangladeshi adults [29]. We estimated that this effect size would be similar in patients with knowlesi malaria of any severity, given the greater haemolysis that occurs in severe knowlesi malaria compared to severe falciparum malaria, and in non-severe knowlesi malaria compared to non-severe falciparum malaria [32].

Using the above calculation, a minimum sample size of 324 will be required. Allowing for a loss to follow-up of 10% (primarily due to patients discharged from hospital < 72 h), we will require a minimum of 360 patients enrolled in the study, 180 in each arm.

### Data collection methods

Venous blood and urine will be collected on enrolment. On venous blood, standard haematology and biochemistry (electrolytes, creatinine, bicarbonate, pH, glucose, lactate and liver transaminases) will be performed through the hospital laboratories. Plasma will be separated within 30 min of collection. Both plasma and urine will be stored at –70 °C until transport for analysis.

Serum creatinine will be measured on enrolment (day 0) then 12-hourly until 72 h, then at 1, 2 and 4 weeks. Follow-up creatinine is measured at 1, 3 and 5 years (Architect c4000 Clinical Chemistry Analyzer, Abbot Laboratories, Illinois, USA). Plasma CFHb concentrations will be measured at enrolment, at 12 h and then daily for 72 h. CFHb will be measured by enzyme linked immunosorbent assay (ELISA) using commercially available kits (Bethyl Laboratories Inc., Montgomery, Texas, USA).

Biomarkers of oxidative stress (plasma F_2_-IsoPs and IsoFs) and endothelial activation/WPB release (Ang-2, vWF, P-selectin, osteoprotegerin (OPG)) will be assessed at admission and then daily for 72 h, with markers of endothelial activation also measured at day 28 and at 1 year. Plasma F_2_-IsoPs and IsoFs will be quantified using gas chromatography-mass spectrometry [42, 43]. Plasma CFHb, Ang-2, vWF, P-selectin and OPG will be measured by ELISA (RnD Systems Inc., Minneapolis, USA). Laboratory assessment and biomarkers of AKI, including microscopy, urine haemoglobin and urine and plasma neutrophil gelatinase-associated lipocalin (RnD Systems Inc., Minneapolis, USA), will be measured daily for 72 h. Urine albumin:creatinine ratio will be performed at 0 and 72 h, then at 1, 2 and 4 weeks and 1, 3 and 5 years. Urinalysis will be performed 12-hourly for 72 h. Urinary glycocalyx degradation products will be measured daily for 72 h [44]. Urine will be collected from a voided specimen or urinary catheter and stored without preservative. Polymerase chain reaction (PCR) for *Plasmodium* spp. will be performed at the Sabah State Reference Laboratory [45, 46] or other validated PCR. Paracetamol levels on enrolment and then 6-hourly for 72 h will be quantified using a validated liquid chromatography-tandem mass spectrometry method [47, 48].

Microscopic peripheral blood parasitaemia will be assessed by research microscopists on admission and 6-hourly until two consecutive negative smears are obtained. Asexual parasite counts will be calculated on thick blood smear using the formula: parasite count per μL blood = parasites × total white blood cell count (from the matched daily automated white blood cell count)/200 white blood cells counted.

G6PD genotyping will be performed on all enrolled patients, as G6PD status may influence oxidative stress and may thus affect the beneficial effects of paracetamol.

### Primary outcomes

- The change in log-transformed creatinine from enrolment (hour 0) to 72 h.
- The change in log-transformed creatinine from enrolment (hour 0) to 72 h stratified by the presence of haemolysis, as measured by CFHb (defined by using a receiver-operating curve analysis to determine the cut-off CFHb that provides maximal sensitivity/specificity for predicting AKI by KDIGO criteria).

### Secondary outcomes

- Longitudinal change in creatinine over 72 h, as measured by the area under the creatinine-time curve, and the effect of enrolment CFHb on the longitudinal change in creatinine.
- Longitudinal change in creatinine over 72 h, as measured by the area under the creatinine-time curve, and the effect of enrolment CFHb on the longitudinal change in creatinine in patients with severe knowlesi malaria and in those with AKI (see Table 1: Criteria for severe *P. knowlesi* malaria).
- Development of AKI (as defined by KDIGO criteria) at 72 h.
- Duration of AKI: Length of time elapsed until serum creatinine returns to measured baseline or if unavailable, presumed basline (estimated by back-calculation of creatinine using the Modification of Diet in Renal Disease equation) in the absence of renal replacement therapy.
- Longitudinal changes in CFHb and markers of oxidative stress (plasma F2-IsoP and IsoFs) over 72 h.
- Longitudinal changes in markers of endothelial activation/WPB release (Ang-2, vWF, P-selectin, OPG) over 72 h.
- Longitudinal changes in markers of endothelial dysfunction as measured by degradation products of the endothelial glycocalyx over 72 h.
- Longitudinal changes in blood and urine biomarkers of AKI over 72 h, including neutrophil gelatinase-associated lipocalin and urine albumin:creatinine ratio.
- Fever clearance time (FCT): time taken for aural temperature to fall below 37.5 °C (FCT-A) and time taken for temperature to fall below 37.5 °C and remain there for at least 24 h (FCT-B).
- Parasite clearance time: Time from commencement of antimalarial treatment to the first of two consecutive negative blood films, with blood films assessed by microscopy every 6 h, and parasite half-life [49].
- Safety of paracetamol in severe and uncomplicated knowlesi malaria, as assessed by the number of patients with adverse events or severe adverse events (SAEs)

**Table 1:** Criteria for severe *Plasmodium knowlesi* malaria

Unrousable coma^a^	Glasgow coma scale < 11
Respiratory distress	Oxygen saturation < 92% with respiratory rate > 30 breaths/min
Shock	Systolic blood pressure < 80 mmHg with cool peripheries or impaired capillary refill
Jaundice	Bilirubin > 50 μmol/L, with parasitaemia > 20,000/μL and/or creatinine > 132 μmol/L
Severe anaemia	Haemoglobin < 7.0 g/dL (adults) Haemoglobin < 5.0 g/dL (children)^a^
Significant abnormal bleeding	Including recurrent or prolonged bleeding (from the nose, gums or venipuncture sites), haematemesis or melena
Hypoglycaemia	Blood glucose < 2.2 mmol/L
Metabolic acidosis	Bicarbonate < 15 mmol/L or lactate > 5 mmol/L
Acute kidney injury	Creatinine > 265 μmol/L
Hyperparasitaemia	Parasite count > 100,000/μL (or > 2% infected red blood cells)

^a^Not reported to date in *P. knowlesi* malaria

### Participant timeline

**Fig. 2:**
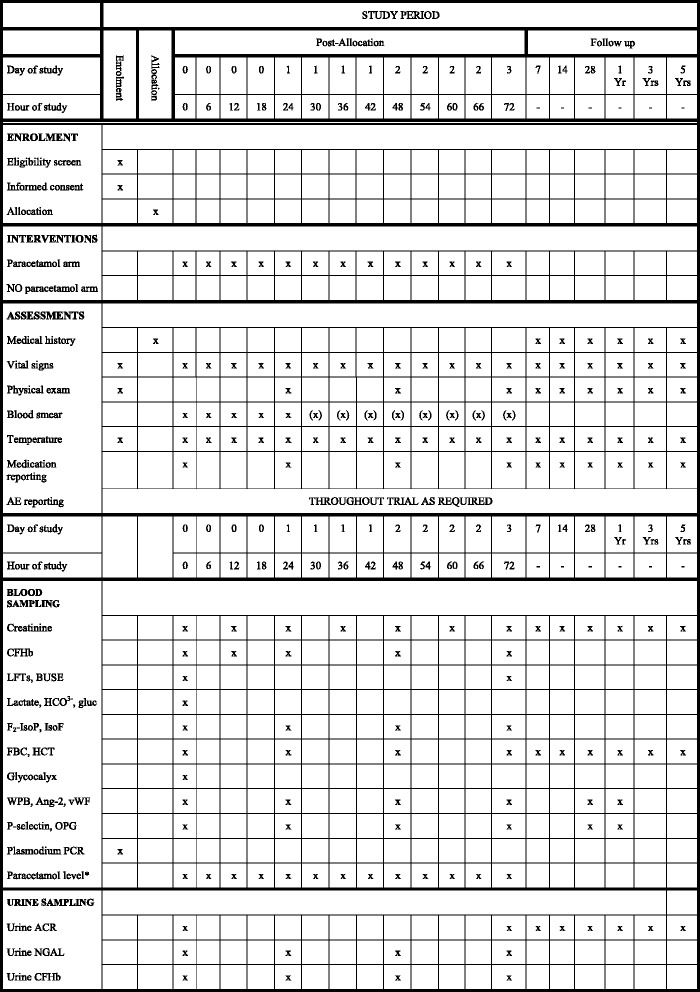
Participant timeline (SPIRIT figure). (x) = Patients without parasite clearance. * Intensive paracetamol level sampling will be performed on a subset of patients at 0.5, 1.5, 2.5, 4.0, 72.5, 73.5, 74.5 and 76.0 h, in addition to 6-hourly as above. *AE* adverse event, *BUSE* blood urea and serum electrolytes, *LFTs* liver function tests (including bilirubin), *CFHb* cell-free haemoglobin, *FBC* full blood count, *HCT* haematocrit, *F*_*2*_*-IsoP* F_2_-isoprostanes, *F*_*2*_*-IsoF* F_2_-isofurans, *WPB* Weibel–Palade bodies, *Ang-2* angiopoietin-2, *vWF* von-Willebrand factor, *OPG* osteoprotegerin, *ACR* albumin:creatinine ratio, *NGAL* neutrophil gelatinase-associated lipocalin

See Fig. 2 – Participant timeline (SPIRIT figure).

## Statistical analysis plan

### Primary endpoint

The primary endpoint will be analysed using ANCOVA with enrolment creatinine (hour 0) as a covariate. The same analysis will be performed in patients stratified by the presence or absence of significant haemolysis. Clinically significant haemolysis will be defined by using a receiver-operating curve analysis to determine the cut-off CFHb that provides maximal sensitivity/specificity for predicting AKI (KDIGO criteria). Analysis of the primary end-point will be by modified intention-to-treat, with patients’ data excluded from the primary analysis if enrolment (hour 0) or 72 h creatinine is unavailable. Patients receiving haemodialysis will be categorised as having had a creatinine rise of 132.6 μmol/L [50]. Based on existing local data in Sabah, this is only expected to be a small proportion of patients (< 5%).

Patients will be excluded if PCR confirms any *Plasmodium* infection other than *P. knowlesi*. In addition, patient data will be excluded from the primary analysis if either 0 or 72 h creatinine measures are not available. For patients with no 72 h creatinine result excluded in the primary analysis, any data collected may still be used in secondary analyses. Patients may voluntarily withdraw from the study at any point for any reason (see below); however, data collected to the point of withdrawal may be used in the relevant analyses.

### Secondary endpoints

The secondary area under the curve endpoints will be compared by Student’s *t* test or Wilcoxon rank-sum test, depending on distribution. Longitudinal data will be analysed using mixed-effects modelling. The parameters of primary interest will be the fixed effect interaction terms between treatment group and times, and treatment group and CFHb at enrolment, to describe whether change in secondary outcomes differs between the two groups across the observation period, and whether the treatment effect depends on enrolment CFHb, respectively. Time-to-event analyses will be used to calculate duration of AKI, fever clearance time and parasite clearance time.

No interim analysis will be performed.

### Additional analyses

1. The effect of paracetamol on the change in creatinine at 72 h and longitudinal change in creatinine over 72 h in patients with CKD, compared to patients without CKD.
2. Change in creatinine at 72 h and longitudinal change in creatinine over 72 h in patients with therapeutic concentrations of paracetamol, compared to patients with absent or low concentrations of paracetamol.
3. Population pharmacokinetics properties of paracetamol, including (1) peak plasma concentration (Cmax), (2) time to peak plasma concentration (Tmax) and (3) area under the plasma drug concentration-time curve (area under the curve).
4. Pharmacodynamic effects of paracetamol on creatinine, fever and parasitemia.
5. To assess the effect of intravascular haemolysis (CFHb) on AKI via specific pathophysiological pathways (oxidative stress, endothelial activation), a mediation analysis will be performed. Using regression modelling, the effect of CFHb on AKI mediated by each specific pathway will be assessed using the product of direct effect of CFHb on pathophysiological outcome (oxidative stress, endothelial activation), and direct effect of these pathophysiological outcomes on AKI.

### Other data to be reported

6. A description of all patients screened and reasons for non-inclusion.
7. Clinical, epidemiological and laboratory features of all patients included in the study.
8. The proportion of all patients lost to follow-up or withdrawn, with reasons for withdrawal.
9. The number of patients with de novo CKD, hypertension and proteinuria at 1, 3 and 5 years after enrolment.

## Additional considerations

### Hepatotoxicity

Previous studies of malaria patients treated with standard dosing of paracetamol have shown no evidence of clinically significant paracetamol-related hepatotoxicity [19, 29, 47, 51]. The standard dosing of paracetamol administered in this study is below that known to be associated with significant hepatotoxicity, and is in accordance with the Malaysian National Drug Formulary. All patients receiving paracetamol will be monitored daily for right upper quadrant pain and tender hepatomegaly, and aspartate transaminase (AST) levels will be measured on admission, at 72 h and on recovery. Additional alanine aminotransferase (ALT) measurements may be collected at the discretion of the treating clinician. If symptoms or signs of hepatotoxicity are present, or there is a rising AST, comprehensive investigations including total bilirubin, International Normalized Ratio and creatinine will be performed. If there is evidence of severe hepatic toxicity (peak AST or ALT levels > 10 times the upper limit of normal), paracetamol administration will be stopped and the patient referred to the gastroenterology consultation service for consideration of N-acetylcysteine therapy.

### Adverse events/termination of the trial

All adverse and SAEs will be recorded as per standard reporting guidelines. SAEs will be reported within 1 day of awareness by the site investigator to the chair of the Safety Monitoring Committee. The Principal Investigator will report the SAE to Malaysian and Menzies ethics committee in accordance with local requirements. Any serious safety concerns identified by the Safety Monitoring Committee may result in modification or termination of the study as necessary.

### Removal of patients from trial

Each participant has the right to withdraw from the study at any time. Additionally, any patient found to have an ALT/AST of 10 times the upper limit of normal will be withdrawn from the study.

The site investigator may also withdraw a participant if the treating clinician determines that further administration of paracetamol is contraindicated for any medical reason. Withdrawn patients will continue to receive standard care from the hospital clinician. The reason for withdrawal will be recorded in the CRF, and any data obtained from the participant up to the time of withdrawal from the study will be included in the analysis. Withdrawn subjects will be replaced.

### Data management

Clinical, biochemical and parasitological information will be collected and managed using REDCap electronic data capture tools hosted at Menzies School of Health Research, Darwin [40]. Analysis will be conducted using STATA, V.14 (StataCorp Ltd., Texas, USA). Data collection will be performed by the study nurses and study physician. An onsite data manager will oversee accuracy and completeness of data entry. The REDCap CRF will include all parameters necessary to assess endpoints.

### Reimbursement for transport

Participants in the study will be reimbursed for their transport to attend all follow-up visits to the study sites. Patients will be paid MYR45 for each day of travelling. No other gifts or payments will be made.

### Confidentiality

All patient information will remain confidential and be shared only by the study team. Unique identifiers are used for computer-based data entry and blood samples. In all cases, the Principal Investigator will ensure that screening forms, CRFs and the completed identification code list are kept in locked files. Subject confidentiality will be protected in any dissemination of results.

## Discussion

This study will be the largest clinical trial to date in knowlesi malaria, and is powered to detect a difference between the two treatment arms in a clinically relevant population including adults and children with knowlesi malaria of any severity. Paracetamol is widely available and has an excellent safety profile; if a renoprotective effect is demonstrated, this trial will support the administration of regularly dosed paracetamol to all patients with knowlesi malaria. The secondary outcomes in this study will provide further insights into the pathophysiology of haemolysis-induced oxidative damage and acute kidney injury in knowlesi malaria, and other haemolytic diseases.

The lack of blinding of study arm randomisation to patients and the treating team is not expected to influence the primary endpoint of change in creatinine, as this is entirely objective. The laboratory staff measuring and reporting creatinine are blinded to treatment allocation. All primary and secondary endpoints listed above are laboratory-based parameters, and therefore not able to be influenced by subjective factors.

All patients will receive standard artemisinin-based therapy as per Malaysian Ministry of Health guidelines and treating clinicians are independent of the study (Additional file 1).

## Trial status

The trial began recruitment on October 18, 2016 and is expected to complete in September 2018.

## Additional file

Additional file 1: Populated SPIRIT checklist. (DOC 121 kb)

## Abbreviations

AKI: Acute kidney injury
ALT: Alanine aminotransferase
Ang-2: Angiopoietin-2
AST: Aspartate transaminase
CFHb: Cell-free haemoglobin
CKD: Chronic kidney disease
CRF: Case report form
F_2_-IsoPs: F_2_-Isoprostanes
FCT: Fever clearance time
IsoFs: Isofurans
KDIGO: Kidney Disease: Improving Global Outcomes
NOX: NADPH oxidase
OPG: Osteoprotegerin
PCR: Polymerase chain reaction
SAE: Serious adverse event
SPIRIT: Standard Protocol Items: Recommendations For Interventional Trials
vWF: von-Willebrand factor
WPB: Weibel–Palade bodies

## Footnotes

1 *P. knowlesi* now accounts for over 90% of all reported malaria cases in Malaysia [4, 6, 37, 38]; however, microscopy is unreliable for distinguishing *P. knowlesi* from *P. falciparum* and *P. vivax* [39]. For blood films reported as *P. falciparum*, a *Pf-*HRP2-based rapid diagnostic test (First Response™; Premier Medical Corp. Ltd., India) is performed, and patients with a positive result are excluded. Patients with nominal *P. vivax* on microscopy are enrolled. Final species confirmation by PCR is performed on all patients; any patient with malaria due to a *Plasmodium* species other than *P. knowlesi* (including mixed *P. knowlesi* infections) will be excluded from all analyses.

2 Pregnant women are excluded due to uncertainty regarding potential adverse effects of untreated fever in pregnancy.
